# Multiple quantum interrogation to determine the position of an object in a serial array of ring resonators

**DOI:** 10.1038/s41598-023-35035-5

**Published:** 2023-05-19

**Authors:** Keigo Nakamura, Daiki Sugio, Takahiro Manabe, Akari Kageyama, Takahiro Matsumoto, Makoto Tomita

**Affiliations:** 1 Department of Physics, Faculty of Science, Shizuoka University, 836, Ohya, Suruga-Ku, Shizuoka, 422-8529 Japan; 2 Graduated School of Design and Architecture, Nagoya City University, Nagoya, 464-0083 Japan

**Keywords:** Optical physics, Optical physics

## Abstract

We propose quantum interaction-free measurements to determine not only whether an object exists, but also where it is situated among possible interrogation positions. In the first configuration, the object exists at one of several possible positions, and the other positions are empty. We regard this as multiple quantum trap interrogation. In the second configuration, the object does not exist in any possible interrogation position, but other positions are occupied by objects. We refer to this as multiple quantum loophole interrogation. It is possible to determine the position of a trap or loophole with almost 100% certainty, without any real interaction between the photon and the objects. We performed a preliminary experiment using a serial array of add-drop ring resonators and confirmed that multiple trap and loophole interrogations are possible. We discuss the detuning of resonators from the critical coupling condition, the loss effects in the resonator, the frequency detuning effect of incident light, and the effect of object semitransparency on the interrogation systems.

## Introduction

Quantum mechanical interaction-free measurements, also known as quantum interrogation, permit optical detection of an absorbing object, despite a lack of photon absorption^1–7^. The original measurement was proposed by Elitzur and Vaidman, based on a Mach–Zehnder interferometer^1^ aligned such that one exit port was dark. At this port, no photon was detected due to complete destructive interference. When an absorbing object was placed in one interferometer path, a photon appeared at the dark detector with a specific probability because destructive interference was broken by the object. Only one photon was injected through the input port. Accordingly, detection of the photon at an exit port indicated that the photon was not absorbed by the object. It is thus possible to obtain knowledge regarding the existence of an object without any real interaction between the photon and the object. The maximum success rate of the original interaction-free measurement was 0.25^1^. This efficiency improved considerably using the quantum Zeno effect^3^ in which the experimental success rate of interaction-free measurements has attained 73% efficiency^4^, which is much higher than the theoretical limit of the original interaction-free measurement. Such measurements have been developed to allow for interaction-free imaging. Optical images of objects have been obtained without the absorption of a photon or with ultra-low intensity light^8–10^. Interaction-free measurements may be used to image biological samples that are sensitive to strong light. Additionally, interaction-free measurements also play a role in counterfactual cryptography, in which some propose that no qubit is required to move between ‘Alice’ and ‘Bob’ when fabricating quantum cryptography keys^11–13^. Notably, counterfactual computation sometimes yields results before the computer is switched on^14–16^. From a practical perspective, an on-chip photonic device based on silicon lithographic technology has been described^17^. Extensions of interaction-free measurements to the electron transport systems of solid-state nanodevices have also been studied^18,19^. All techniques exploit the principle that the presence of an absorbing object destroys interference, even if no particle is absorbed by that object.

In the conventional quantum interrogation systems investigated to date, there is only one interrogation position. This is true even in interaction-free imaging^8–10^. As in these imaging processes, independent interaction-free measurements are performed for each individual image pixel via repeat translation of the single-photon detector across the image. Here, we present a novel quantum interaction-free measurement method that reveals the position of the object. That is, the measurement determines not only whether the object exists or not, but also where the object is situated among multiple possible interrogation positions.

**Figure 1:**
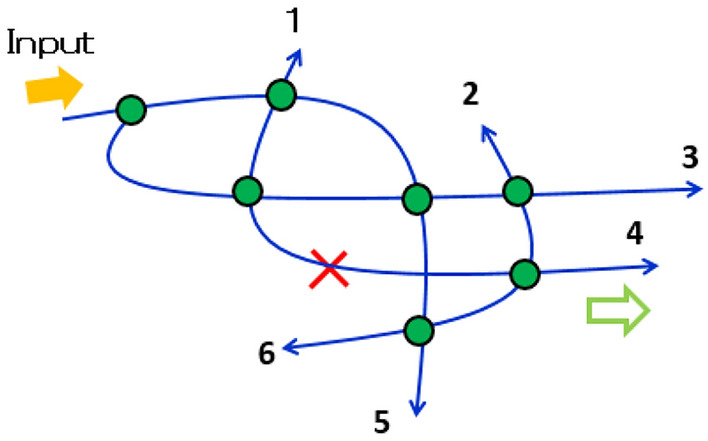
Concept of multiple quantum interrogation. Blue lines are optical paths; solid green circles indicate beam couplers that mix the light fields in the optical paths. A single photon is injected through the input port; ports 1–6 are photon detection ports. The red cross indicates one possible position of an object. Initially (i.e., without the object), some detection ports are dark due to destructive interference among possible optical paths.

Figure 1 illustrates the principle. Initially (when objects are absent), some exit ports are dark because of the destructive interference among the many possible optical paths. When an object exists, the destructive interference is broken and a photon appears at a specific exit port (for example, port 4 in Fig. 1) with a specific probability. If the photon-detecting ports can be correlated with the object positions, the object location can be determined. Because only one photon is injected into the input port, the detection of a photon at a detection port indicates that the measurement proceeded without any real interaction. Here, we focus on a serial array of add-drop ring resonators. Sharp lines in ring resonators and microrings have a variety of potential sensing applications^20^. In the first configuration, we consider a situation in which the object exists in one possible interrogation position, but the other positions are empty. We regard this as multiple quantum trap interrogation. In the second configuration, the object does not exist in any possible interrogation position, but the other positions are occupied by objects. This is known as multiple quantum loophole interrogation. When a single photon is injected into the input port, it is possible to learn (with ~ 100% certainty) the position of the trap or loophole, without the requirement for real interactions between the photon and any object. Using the dynamic recurrent loop system discussed in “Experiments involving multiple interrogation at the classical intensity level” section, we performed preliminary experiments with classical optical pulses showing that multiple trap interrogation and loophole interrogation are possible. We discuss detuning of the resonator from the critical coupling condition, losses in the resonator, the frequency detuning effect of the incident light, and the effects of semitransparent absorbing objects on the interrogation systems.

## Configurations for multiple quantum interrogation

**Figure 2:**
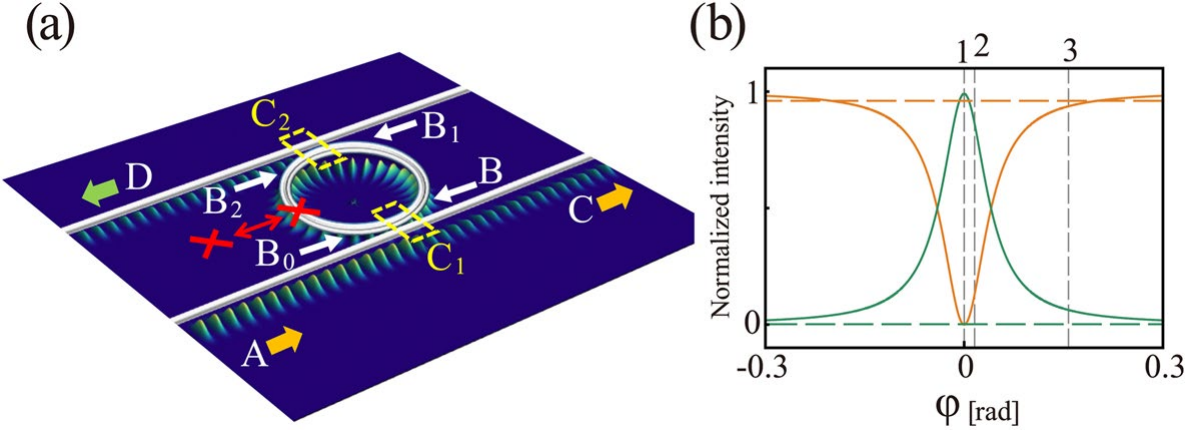
(**a**) Schematic diagram of the add-drop ring resonator. The white lines and circle represent the waveguide and ring resonator, respectively, and the red cross indicates the interrogation object. C_1_ and C_2_ are couplers. A, input port. C, D, output ports. B, B_1_, B_2_, and B_0_ are the positions where the electric field is considered. (**b**) Typical transmission spectra through the add-drop ring resonator as a function of the detuning frequency calculated using analytical equation (Eq. 3) for $x_{1}$ = 0.9999, $x_{2}$ = 0.9999, $y_{1} = x_{1} x_{2} y_{2}$ (0.9798), and $y_{2}$ = 0.9800. Orange and green lines refer to the output ports C (through port) and D (drop port), respectively. Solid lines refer to an empty resonator, and dashed lines refer to a resonator with an object. The vertical dashed lines represent the resonance conditions. Line 1 is the on-resonance condition, and lines 2 and 3 are the off-resonance conditions ($\varphi = 0,\pi /200$ and $\pi /20$, respectively). (**a** was created using Wolfram Mathematica 12).

We assume an optical add-drop ring resonator (Fig. 2)^21,22^. To evaluate the probability of photon detection, we must link the creation and annihilation operators of the output ports to the corresponding operators of the input port. This linkage is achieved by modifying the annihilation operators as given below^23^:

1 $$\begin{aligned} \hat{a}_{C} & = (1 - \gamma_{1} )^{\frac{1}{2}} \left[ {\hat{a}_{A} \cos (\xi_{1} ) - i\hat{a}_{{B_{0} }} \sin (\xi_{1} )} \right] \\ \hat{a}_{B} & = (1 - \gamma_{1} )^{\frac{1}{2}} \left[ { - i\hat{a}_{A} \sin (\xi_{1} ) + \hat{a}_{{B_{0} }} \cos (\xi_{1} )} \right] \\ \hat{a}_{{B_{1} }} & = \hat{a}_{B} \exp ( - \frac{\rho }{4} + i\beta \frac{L}{2}) \\ \hat{a}_{{B_{0} }} & = \hat{a}_{{B_{2} }} T_{\kappa } \exp ( - \frac{\rho }{4} + i\beta \frac{L}{2}) \\ \hat{a}_{D} & = (1 - \gamma_{2} )^{\frac{1}{2}} \left[ { - i\hat{a}_{{B_{1} }} \sin (\xi_{2} )} \right] \\ \hat{a}_{{B_{2} }} & = (1 - \gamma_{2} )^{\frac{1}{2}} \left[ {\hat{a}_{{B_{1} }} \cos (\xi_{2} )} \right] \\ \end{aligned}$$

Each operator $\hat{a}_{j}$ (where $j$ = $A$, $B$, $B_{0}$, $B_{1}$, $C$, and $D$) is an annihilation operator at the positions shown in Fig. 2a, $\rho$ is the extinction within the ring resonator, and $\gamma_{i}$( $i$ = 1, 2) is the insertion loss at couplers C_1_ and C_2_, respectively. We introduce the notation $x_{i} \equiv (1 - \gamma_{i} )^{\frac{1}{2}} \exp ( - \rho /4)$, $y_{i} \equiv \cos (\xi_{i} )$, ($i$ = 1, 2), where $\xi_{i}$($i$ = 1, 2) is the interaction length at couplers C_1_ and C_2_, respectively. The product of the loss parameters $x_{1} x_{2}$ ($0 \le x_{1} x_{2} \le 1$) represents attenuation of the electromagnetic field after one round trip in the ring resonator. Parameter $y_{i}$ ($0 \le y_{i} \le 1$) is the coupling parameter, which is the electric field transmittance through couplers C_1_ and C_2_, respectively. The phasor is given by $e^{i\varphi (\nu )} \equiv \exp (i\beta (\nu )L)$, where $\varphi (\nu )$ is the phase shift in the circulation orbit in the ring resonator, and $\beta (\nu )$ and $L$ are the resonator propagation constant and optical path length, respectively. $T_{\kappa }$ is the field transmittance of the object ($0 \le T_{\kappa } \le 1$). In this section, we consider a perfectly absorbing object: $T_{\kappa }$ = 0. When $T_{\kappa }$ = 1, the object is perfectly transparent or absent from the resonator. Using Eq. (1), $\hat{a}_{C}$ and $\hat{a}_{D}$ become

2 $$\begin{aligned} \hat{a}_{C} & = C(x_{1} ,x_{2} ,y_{1} ,y_{2} ,T_{\kappa } ,\varphi )\;\hat{a}_{A} \\ \hat{a}_{D} & = D(x_{1} ,x_{2} ,y_{1} ,y_{2} ,T_{\kappa } ,\varphi )\;\hat{a}_{A} \\ \end{aligned}$$

where

3 $$\begin{gathered} C(x_{1} ,x_{2} ,y_{1} ,y_{2} ,T_{\kappa } ,\varphi ) = (1 - \gamma_{1} )^{\frac{1}{2}} \left[ {\frac{{y_{1} - T_{\kappa } x_{1} x_{2} y_{2} e^{ + i\varphi } }}{{1 - T_{\kappa } x_{1} x_{2} y_{2} y_{1} e^{ + i\varphi } }}} \right] \hfill \\ D(x_{1} ,x_{2} ,y_{1} ,y_{2} ,T_{\kappa } ,\varphi ) = \frac{{(1 - \gamma_{2} )^{\frac{1}{2}} x_{1} \sqrt {1 - y_{1}^{2} } \sqrt {1 - y_{2}^{2} } e^{{ + i\frac{3}{2}\varphi }} }}{{T_{\kappa } x_{1} x_{2} y_{1} y_{2} - 1}} \hfill \\ \end{gathered}$$

In the experiments, a single photon is injected through the input port:

$$< 1_{A} |\hat{a}_{A}^{ + } \hat{a}_{A} |1_{A} > = 1,$$

where $|1_{A} >$ denotes the single photon state at the input mode. The respective probabilities that the photon is detected at ports C (through port) and D (drop port) are given by

4 $$\begin{gathered} P_{j} (C) = < 1_{A} |\hat{a}_{C}^{ + } \hat{a}_{C} |1_{A} > = \left| {C_{j} } \right|^{2} \hfill \\ P_{j} (D) = < 1_{A} |\hat{a}_{D}^{ + } \hat{a}_{D} |1_{A} > = \left| {D_{j} } \right|^{2} \hfill \\ \end{gathered}$$

We use the subscript $j = A$ when the object is absent and $j = P$ when the object is present. Figure 2b shows example $\left| {C_{A} (\varphi )} \right|^{2}$ and $\left| {D_{A} (\varphi )} \right|^{2}$ functions with reference to $\varphi$. At the resonance frequency ($\varphi$ = 0), in the absence of the object, the incident photon is detected at port D with high probability, such that $P_{A} (D) = \sim 1$ (solid green line in Fig. 2b). The probability of incident photon detection at port C is $P_{A} (C) = 0$ (solid orange line). Conversely, in the presence of the object, the photon is detected at port C with high probability, such that $P_{P} (C) = \sim 1$ (dashed orange line). The probability of photon detection at port D is $P_{P} (D) = \sim 0$ (dashed green line).

### Multiple quantum trap interrogation

**Figure 3:**
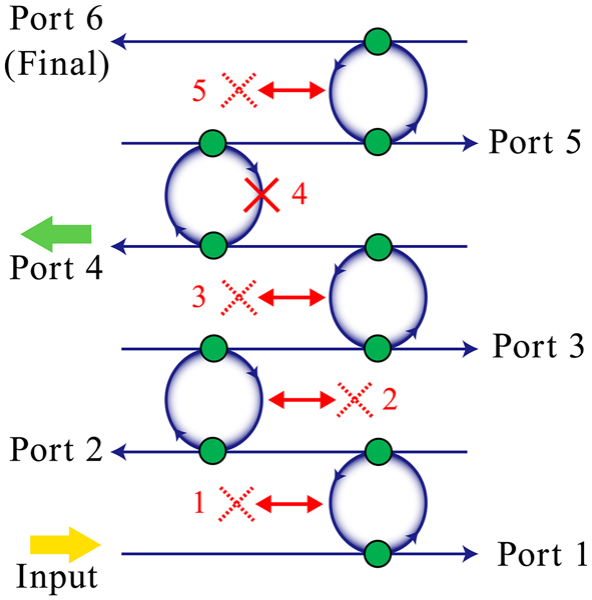
Configuration for multiple quantum trap interrogation. Blue lines and circles represent the bus waveguide and add-drop ring resonators, respectively; green circles represent the couplers. Ports 1–6 have single-photon detectors. Red crosses represent objects; here, the object exists in position 4 and the other positions are empty.

We first consider the scheme shown in Fig. 3. In this configuration, there are $N$ ring resonators and $N + 1$ ports. The object exists at one of the interrogation positions (ring resonators) labeled $m$, where $1 \le m \le N$. A single photon is injected through the input port, thus $<1_{A}|1_{A} >$ = 1. $P_{T}^{{}} (n,m)$ is the probability of photon detection at port $n$ when the object exists in interrogation position $m$, where $1 \le n \le N + 1$ and $1 \le m \le N$. The lower subscript T represents the configuration of quantum multi-trap interrogation. The probability $P_{T}^{{}} (n,m)$ is given by

5 $$\begin{gathered} n \le m - 1 \hfill \\ \begin{array}{*{20}c} {} & {} \\ \end{array} P_{T}^{{}} (n,m) = < 1_{A} |\hat{a}_{n}^{ + } \hat{a}_{n} |1_{A} > = \left| {\left[ {D_{A} } \right]^{n - 1} C_{A} } \right|^{2} \hfill \\ n = m \hfill \\ \begin{array}{*{20}c} {} & {} \\ \end{array} P_{T}^{{}} (n,m) = \left| {\left[ {D_{A} } \right]^{n - 1} C_{P} } \right|^{2} \hfill \\ n \ge m + 1 \hfill \\ \begin{array}{*{20}c} {} & {} \\ \end{array} P_{T}^{{}} (n,m) = \left| {\left[ {D_{A} } \right]^{n - 2} D_{P} C_{A} } \right|^{2} \hfill \\ \begin{array}{*{20}c} {} & {} \\ \end{array} P_{T}^{{}} (n,m) = \left| {\left[ {D_{A} } \right]^{n - 2} D_{P} } \right|^{2} \begin{array}{*{20}c} {} & {} \\ \end{array} {\text{final port}}\begin{array}{*{20}c} {} & {(n = N + 1)} \\ \end{array} \hfill \\ \end{gathered}$$

Under the critical coupling condition, $C_{A}$ = 0, when $\varphi (\nu )$ = 0 (on-resonance frequency condition). Furthermore, for resonators with small losses, $D_{P}$ ~ 0. Hence, the probability is $P_{T}^{{}} (n \ne m,m)$ = 0, which indicates that the photon is not detected at port $n \ne m$.

**Figure 4:**
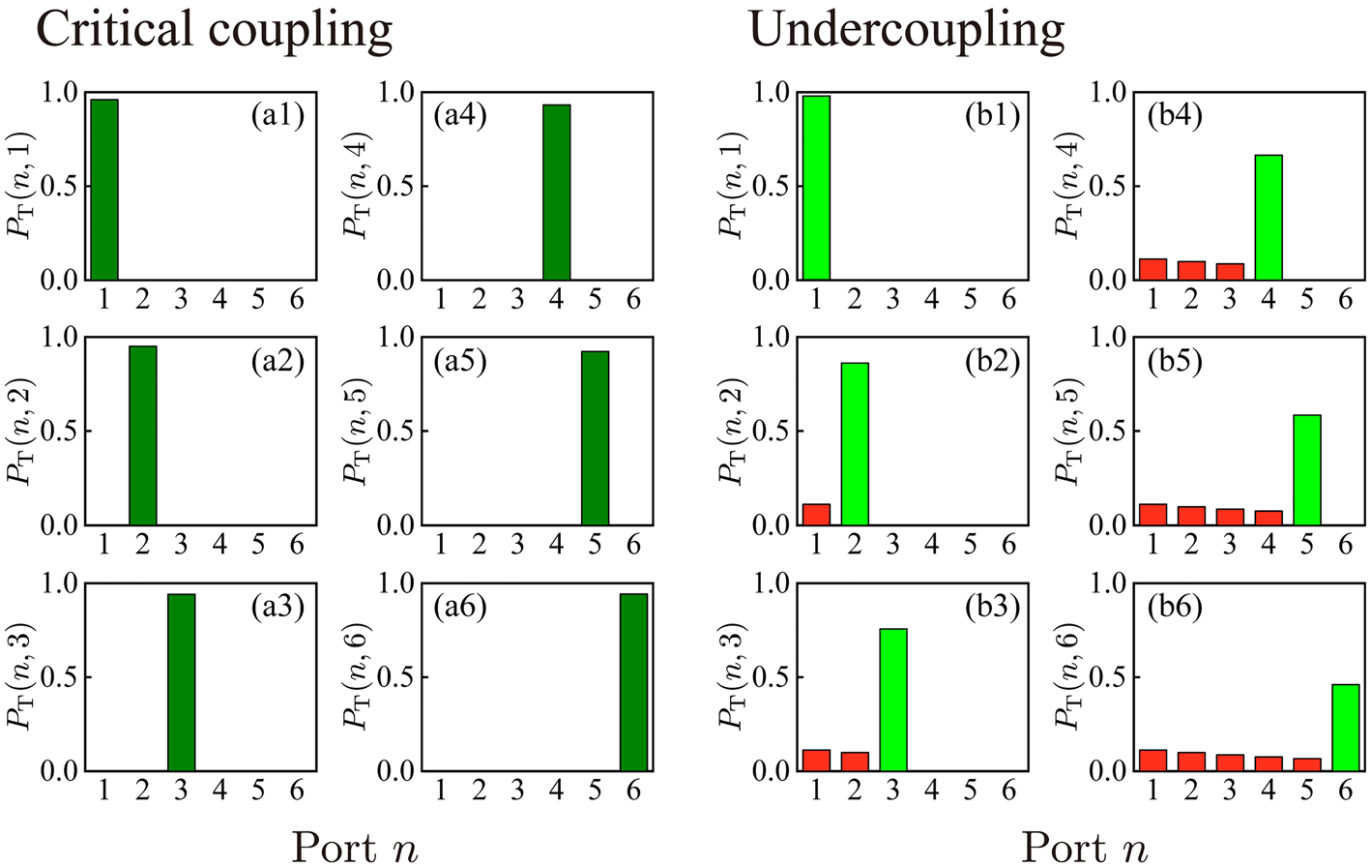
Calculation of photon detection probabilities at each exit port $P_{T}^{{}} (n,m)$ for $N$ = 5. (**a**) Critical coupling: $x_{1}$ = 0.9999, $x_{2}$ = 0.9999, $y_{1} = x_{1} x_{2} y_{2}$(= 0.9798), and $y_{2}$ = 0.9800. (**b**) The resonator is slightly detuned from the critical coupling condition (and is now undercoupled, as discussed in “Coupling conditions, absorption, laser detuning, and semitransparency” section). Here, $y_{1}$ = 0.9898. The other parameters are the same as the parameters in (**a**).

Figure 4(a1–a6) show the photon detection probabilities at each detection port $n$, $P_{T}^{{}} (n,m)$, for $N$ = 5 and $x_{1}$ = 0.9999, $x_{2}$ = 0.9999, $y_{1} = x_{1} x_{2} y_{2}$(= 0.9798), and $y_{2}$ = 0.9800 calculated under the critical coupling condition. Calculations were performed under on-resonance frequency conditions. In Fig. 4(b1–b6), the plots are similar for the undercoupling condition. For example, Fig. 4(a3) depicts the case where the object exists at position $m$ = 3. The detection probability $P_{T}^{{}} (n = 3,m = 3)$ = 0.94, and the probability of photon detection at the other positions is $P_{T}^{{}} (n \ne 3,m = 3)$ = 0. We now discuss the distinguishability $\eta (n,m = n)$, defined as

6 $$\eta_{T \, or \, L}^{{}} (n,m) = \frac{{P_{T \, or \, L}^{{}} (n,m)}}{{\sum\limits_{m^{\prime} = 1}^{N + 1} {} P_{T \, or \, L}^{{}} (n,m^{\prime})}}$$

This is the probability that the object exists at position $m$ when the photon is detected at port $n$. The condition $\eta (n,m)$ = 1 thus indicates that when the photon is detected at port $n$, there is 100% certainty that the object is at $m$. Under the critical coupling condition, $\eta_{T}^{{}} (n,m = n)$ = 1. Therefore, the position of the object can be predicted with 100% certainty, even though the photon never interacted with the object. We regard $P_{T}^{{}} (n,m = n)$ as the success rate, because this is the probability of photon detection at the object position. These two features, i.e., $\eta_{T}^{{}} (n,m = n)$ = 1 and the reasonably high success rate $P_{T}^{{}} (n,m = n)$, form the basis of quantum multiple interrogation.

The physical background of quantum multiple interrogation can be intuitively explained as follows. The output beam at the through port (port C in Fig. 2a) consists of two components: a ballistic component that directly appears at port C, bypassing the ring resonator, and a circulated component that travels around the ring repeatedly after entering the ring resonator, and exits at port C via the coupler. These two components are $\pi$ rad phase-shifted with respect to each other, which results in destructive interference. Under the critical coupling condition, the amplitude of the two components are equal, and the output at port C is zero due to the destructive interference. This situation is the same as the original interaction-free measurement using a Mach-Zehnder interferometer proposed by Elitzur and Vaidman. The high efficiency of the present interaction-free measurement compared with the results of Elitzur and Vaidman is attributed to the high finesse of the interferometer^6^. The advantage of the add-drop ring resonator is the existence of the drop port (port D in Fig. 2a): when the object is absent from the ring, the circulated light interferes and almost all of the photon appear at port D when the loss in the ring is negligible. Then, the surviving photon that completed the first-stage interrogation can go to the next stage. A similar configuration can be obtained using a serial array of all pass ring resonators. However, in this case, the photon will be absorbed within the ring resonator when the object does not exist. Hence, a multiple interrogation configuration cannot be obtained.

### Multiple quantum loophole interrogation

**Figure 5:**
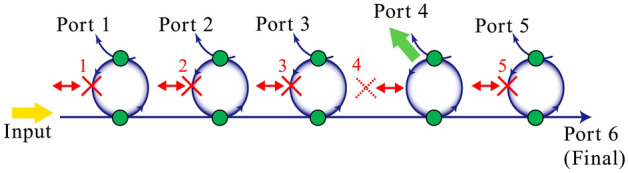
Configuration for multiple quantum loophole interrogation. Here, interrogation position 4 is empty, but the other positions are occupied by objects.

We next consider the scheme of Fig. 5. In this case, objects exist at all possible positions except one, which is labeled $m$. A single photon is injected through the input port and detected at port $n$ where $1 \le n \le N + 1$. If the object is an ultrasensitive bomb, we must identify a safe path among $N$ possible paths without real interaction. The probability of photon detection at position $n$, $P_{L}^{{}} (n,m)$, is

7 $$\begin{gathered} n \le m - 1 \hfill \\ \begin{array}{*{20}c} {} & {} \\ \end{array} P_{L}^{{}} (n,m) = < 1_{A} |\hat{a}_{n}^{ + } \hat{a}_{n} |1_{A} > = \left| {\left[ {C_{P} } \right]^{n - 1} D_{P} } \right|^{2} \hfill \\ n = m \hfill \\ \begin{array}{*{20}c} {} & {} \\ \end{array} P_{L}^{{}} (n,m) = \left| {\left[ {C_{P} } \right]^{n - 1} D_{A} } \right|^{2} \hfill \\ n \ge m + 1 \hfill \\ \begin{array}{*{20}c} {} & {} \\ \end{array} P_{L}^{{}} (n,m) = \left| {\left[ {C_{P} } \right]^{n - 2} C_{A} D_{P} } \right|^{2} \hfill \\ \begin{array}{*{20}c} {} & {} \\ \end{array} P_{L}^{{}} (n,m) = \left| {\left[ {C_{P} } \right]^{n - 2} C_{A} } \right|^{2} \begin{array}{*{20}c} {} & {} \\ \end{array} {\text{final port}}\begin{array}{*{20}c} {} & {(n = N + 1)} \\ \end{array} \hfill \\ \end{gathered}$$

For a perfectly absorbing object and resonators with small intrinsic losses operating under critical coupling, the probability $P_{T}^{{}} (n \ne m,m)$ =  ~ 0. Therefore, the distinguishability of the object position is ~ 100%. Accordingly, the presence of the photon at port $n$ indicates (with ~ 100% certainty) that a loophole exists at $n$. No real interactions are needed to support this conclusion. The distinguishability is ~ 1, even when the number of ring resonators increases, as long as the resonators operate under the critical coupling condition.

## Coupling conditions, absorption, laser detuning, and semitransparency

**Figure 6:**
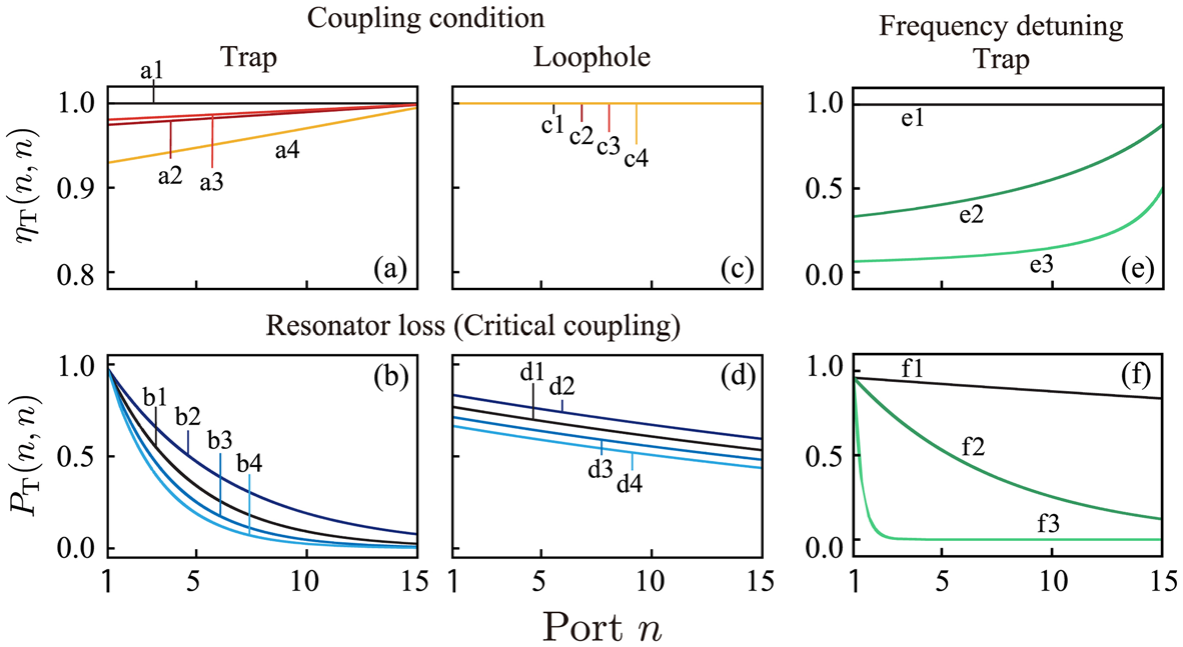
(**a**) Distinguishability curves $\eta_{T} (n,m = n)$ under different coupling conditions for multiple quantum trap interrogation. Curve (a1) is the curve under critical coupling. Curve (a2) is the curve for undercoupling, and curves (a3) and (a4) are the curves for overcoupling. Parameter used for curves are $x_{1}$ = (a1) 0.9980 ($10Log_{10} (x_{1} x_{2} ) =$ − 1.3 × 10^−2^ dB), (a2) 0.9990 ($10Log_{10} (x_{1} x_{2} ) =$ − 8.7 × 10^−3^ dB), (a3) 0.9970 ($10Log_{10} (x_{1} x_{2} ) =$ − 1.7 × 10^−2^ dB), and (a4) 0.9960 ($10Log_{10} (x_{1} x_{2} ) =$ − 2.2 × 10^−2^ dB). Other parameters are $x_{2}$ = 0.9990, $y_{1}$ = 0.9870, and $y_{2}$ = 0.9900 for all curves. (**b**) Curves of $P_{T}^{{}} (n,m = n)$ at different losses. All curves were derived under critical coupling, i.e.$y_{1} = x_{1} x_{2} y_{2}$. The parameters $x_{1}$ used for curves (b1–b4) are the same as those used for (a1–a4), respectively. Other parameters are $x_{2}$ = 0.9990, and $y_{2}$ = 0.9900 for all curves. (All parameters $x_{1}$,$x_{2}$,$y_{1}$ and $y_{2}$ used for (a1), (b1), (c1) and (d1) are same; black curves). (**c**,**d**) Distinguishability curves of $\eta_{L} (n,m = n)$ and $P_{L}^{{}} (n,m = n)$ for multiple quantum loophole interrogation under different coupling conditions. The parameters used in (c1–c4) and (d1–d4) are the same as those used in (a1–a4) and (b1–b4), respectively. (**e**,**f**) The distinguishability curves are $\eta_{T} (n,m = n)$ and $P_{T}^{{}} (n,m = n)$ for multiple quantum trap interrogation under laser detuning conditions. Parameter used are the same as those used in Fig. 2b ($x_{1}$ = 0.9999. $x_{2}$ = 0.9999, $y_{1} = x_{1} x_{2} y_{2}$(= 0.9798) and $y_{2}$ = 0.9800). The frequency detuning conditions are $\varphi=$ (e1,f1) 0, (e2,f2)$\pi /200$ rad, and (e3,f3)$\pi /20$ rad.

We consider factors that affect multiple interrogation. First, we discuss detuning of the resonators from the critical coupling condition. Figure 6a shows the distinguishability $\eta_{T}^{{}} (n,m = n)$ under different coupling conditions. Calculations were carried out under on-resonance frequency conditions. Curve a1 is the curve for critical coupling. Under this condition, $\eta_{T}^{{}} (n,m = n)$ = 1. Therefore, the position of the object can be predicted with 100% certainty. Curve a2 is the curve for the undercoupling condition. Curves a3 and a4 are the curves for overcoupling conditions. Under non-critical (under- or over-) coupling conditions, the distinguishability decreases below unity and quantum interrogation is imperfect. The explanation is clear from Fig. 4(b1–b6), which shows the photon detection probabilities $P_{T}^{{}} (n,m)$ in the undercoupling condition. As indicated by the red histogram in Fig. 4b, the photon is detected at ports $n < m$; therefore, photon detection at a specific port $n$ does not necessarily indicate that the object exists at position $m = n$. This reduces the distinguishability $\eta_{T}^{{}} (n,m = n)$. Additionally, the distinguishability $\eta_{T}^{{}} (n,m = n)$ under the non-critical coupling conditions of Fig. 6a (curves a2, a3, and a4) increases as a function of $n$. For a perfectly absorbing object, its existence at position $m$ indicates that the photon detection probability at port $n$; $n > m$ is very small. Therefore, photon detection at port $n$ excludes the possibility that the object exists at positions $m < n$. This result increases the distinguishability $\eta_{T}^{{}} (n,m = n)$ as $n$ increases.

Next, we consider the effect of resonator loss on the success rate $P_{T}^{{}} (n,m = n)$. Figure 6b shows the $P_{T}^{{}} (n,m = n)$ values for the different loss parameters $x_{1}$. Calculations were performed under on-resonance frequency conditions. For all curves, we set the coupling parameter $y_{1}$ to $y_{1} = x_{1} x_{2} y_{2}$, and then prepared the resonator for critical coupling. As discussed above, the distinguishability is unity for all curves in Fig. 6b under critical coupling. However, for high-performance quantum interrogation, a high success rate is also important. The success rates with large losses (curves b1, b3, and b4 in Fig. 6b) are lower than the success rates with a small loss (curve b2 in Fig. 6b). Furthermore, the success rate $P_{T}^{{}} (n,m = n)$ decreases as $n$ increases. All of these tendencies are explained by photon absorption during propagation.

Similar calculations were performed for multiple quantum loophole interrogation. Figure 6c shows the curves of the distinguishability $\eta_{L}^{{}} (n,m = n)$ under different coupling conditions. The distinguishability $\eta_{L}^{{}} (n,m = n) =$  ~ 1, as all curves nearly overlap. Figure 6d shows the curves of $P_{L}^{{}} (n,m = n)$ under the critical coupling condition for different resonator losses. The success rate $P_{L}^{{}} (n,m = n)$ decreases as the loss increases.

All of the theoretical calculations discussed so far were carried out assuming that the incident laser frequency was resonant on the ring resonators. We now consider the off-resonance effect of the incident laser frequency. The vertical dashed lines in Fig. 2b indicate the light frequency. Line 1 indicates the on-resonance frequency condition and lines 2 and 3 represent the off-resonance condition ($\phi =$ $\pi /200$ rad and $\pi /20$ rad). When the laser frequency is off-resonance, the destructive interference at the through port and constructive interference at the drop are broken. These effects reduce distinguishability. Figure 6e,f show the increase in distinguishability and the reduction detection probability, respectively, as a function of $n$. Multiple quantum interrogations do not work well under the off-resonance frequency condition.

**Figure 7:**
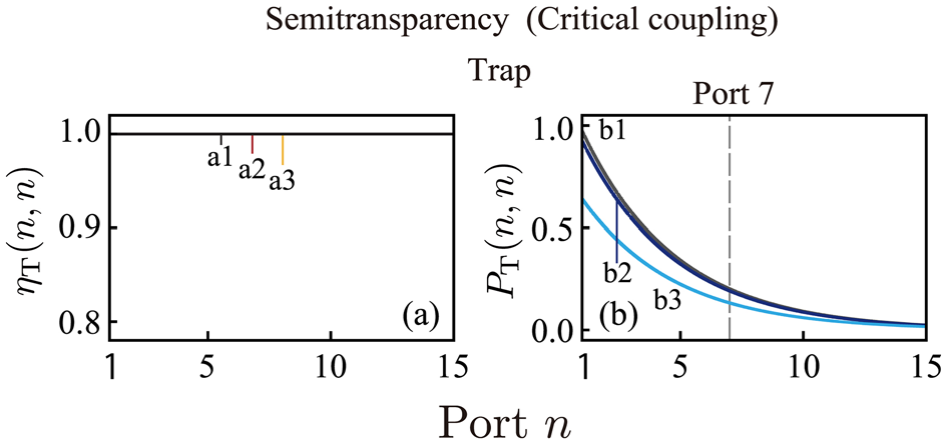
(**a**) Distinguishability $\eta_{T} (n,m = n)$ and (**b**) success rate $P_{T}^{{}} (n,m = n)$ curves at different transmittances of the object $T_{\kappa }$ for multiple quantum trap interrogation under the critical coupling condition. $T_{\kappa }$ = 0, 0.5, and 0.9 for curves 1, 2 and 3, respectively; $x_{1}$ = 0.9980, $x_{2}$ = 0.9990, $y_{1} = x_{1} x_{2} y_{2}$, and $y_{2}$ = 0.9900 for all curves.

Thus far, we have assumed that the object was perfectly absorbing. Next, we discuss the effect of object semitransparency^24,25^ on the distinguishability $\eta_{T}^{{}} (n,m = n)$ and the success rate $P_{T}^{{}} (n,m = n)$. Figure 7a shows $\eta_{T}^{{}} (n,m = n)$ for a semitransparent object for multiple quantum trap interrogation under the critical coupling condition. We denote the object transmittance as $T_{\kappa }$ ($0 \le T_{\kappa } \le 1$) and ignore the phase shift caused by object thickness. Calculations were carried out under the on-resonance frequency condition. The resonators were prepared for critical coupling; thus, $\eta_{T}^{{}} (n,m = n)$ =  ~ 1, independent of $T_{\kappa }$. Notably, in Fig. 7b, $P_{T} (n,m = n)$ decreases only slightly when the transparency of the object increases. For example, $P_{T} (n = 7,m = 7)$ = 0.20 for a perfectly absorbing object (curve b1 in Fig. 7b) and decreases to 0.19 for the semitransparent object of $T_{\kappa }$ = 0.5 (curve b2 in Fig. 7b). That is, the $P_{T} (n,m = n)$ reduction is only 1%, even when the object is 50% transparent. Thus, multiple quantum trap interrogation functions in a robust manner, even for highly transparent objects. The high success rate $P_{T} (n,m = n)$ for a semitransparent object reflects the high value of the coefficient $C_{P} (T_{\kappa } )$ in Eq. (2) for a semitransparent object. The critical coupling condition in a high-Q resonator is sensitive to resonator loss, where even small absorption induced by a semitransparent object breaks the critical coupling. Then, the photon exits via the required port. These results explain why the system functions in a robust manner, even for a highly transparent object.

Here, we ignored the phase shift caused by the object. When this shift is larger than the resonance linewidth of the resonator, the existence of the object breaks the on-resonance condition. Then, the photon appears at the through port after the insertion of the object, which was dark before the insertion. If the object is ultrasensitive to the photon, a similar principle of interaction-free measurements is expected to apply to this object. That is, using a single photon, we can detect the object by the phase shift without real interaction between the photon and object.

## Experiments involving multiple interrogation at the classical intensity level

**Figure 8:**
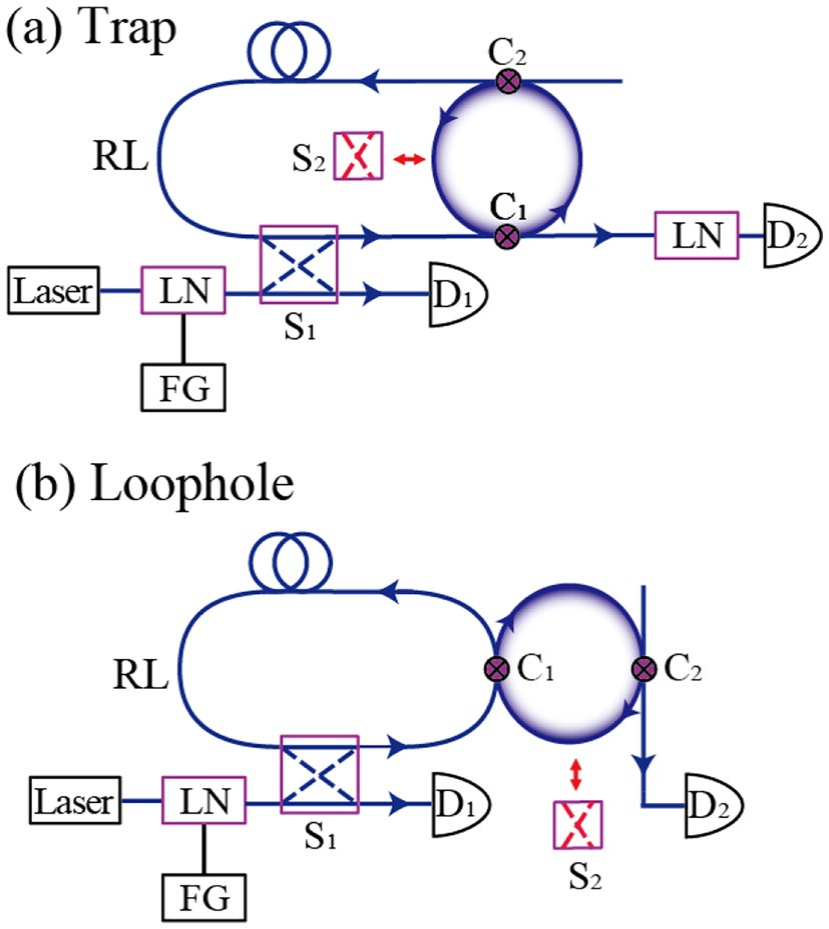
Schematic diagram of the experimental setup based on a time-divided recurrent system. (**a**) Multiple quantum trap interrogation and (**b**) multiple quantum loophole interrogation. *LN*, LiNbO_3_ modulator; *S*_*1*_, 2 × 2 fast optical switches; *S*_*2*_, high-extinction optical switch; *C*_*1*_*, C*_*2*_, couplers; *R*, ring resonator; *RL*, recurrent loop; *D*_*1*_*, D*_*2*_*,* detectors; *FG*, function generator.

In ideal ring resonators with very small losses, both high distinguishability and detection probability can be achieved. In such a system, even when the incident light is not a real single photon state, a principle similar to the interaction-free measurement is expected to work. To explore multiple interrogation, we performed experiments involving multiple trap and loophole interrogations at the classical intensity level. The setup is shown in Fig. 8: we used a dynamic recurrent loop system^26,27^. An Er-fiber laser operating at 1556 nm was used as the incident light source. The spectral width was 1 kHz, and the laser frequency was precisely tuned via piezoelectric control of the cavity length. Gaussian-shaped pulses of duration $t_{p}$ = 110 ns, with a repetition rate of 1.5 kHz, were created using a 240-MHz function generator and LiNbO_3_ modulator. The pulse duration satisfied the condition $t_{p} > \delta \nu^{ - 1}$, where $\delta \nu$ is the resonance band width of the ring resonator. This constraint was needed to allow the resonator to attain the steady state. The laser beam was filter-attenuated and thus delivered weak coherent incident light. The mean power was 10–1000 μW. This pulse contained approximately 10^7^–10^9^ photons. The dynamic recurrent system contained 2 × 2 fast optical switches S_1_ and a polarization-maintenance fiber with optical length $L_{RL}$ = 500 m as the recurrent loop. The rise and fall times of S_1_ were both 50 ns. When the incident optical pulse initially arrived at optical switch S_1_, S_1_ opened at $\Delta \tau$ = 1600 ns and injected the optical pulse into the recurrent loop. Then, S_1_ closed and the pulse was confined within the recurrent loop equipped with the add-drop ring resonator. The add-drop ring resonator was constructed using a polarization-maintaining fiber. Coupler C_2_ was a fixed ratio coupler. We used two types of couplers ($y_{2}$ = 0.995 and 0.74) for C_2_. Coupler C_1_ was a variable coupler that adjusted the intensity branching ratio of the ring resonator^28^. We carefully adjusted the coupling ratio of C_1_ around the value of $y_{1}$ = 0.78 ± 0.1 to achieve the critical coupling condition $T_{Abs} (C)$ = 0, where $T_{Abs} (C)$ is the intensity transmittance at the through port in the absence of the object. For the single-stage ring resonator, the intensity transmittances at the through port in the presence of the object was $T_{Pre} (C)$ = 0.62 and the intensity transmittances at the drop port in the absence of the object was $T_{Abs} (D)$ = 0.22. The loss parameter of the ring resonator was determined as $x_{1} x_{2}$ = 0.78 ($10Log_{10} (x_{1} x_{2} ) = - 1.1\;{\text{dB}}$) by the transmission measurements.

Figure 8a shows the configuration for multiple quantum trap interrogation. The pulse from the drop port of the ring resonator was retuned in the recurrent loop. The pulse repeatedly passed through the ring resonator. As the absorbing object, we inserted a high-extinction ratio optical switch S_2_ (extinction ratio 10^−4^) into the ring resonator. Placement of the object at interrogation position $m$ was achieved by closing S_2_ for $\Delta \tau$ = 1600 ns at time $\tau_{m} = (m - 1)\tau_{0}$, where $\tau_{0} = L_{RL} n_{eff} /c$ = 2500 ns is the trip time of the pulse around the recurrent loop and $n_{eff}$ is the effective refractive index. During other times, S_2_ was open. The pulse was detected by the InGaAs avalanche photodiode D_2_. To observe the photon in the final port ($N + 1$), the switch S_1_ re-opens at time $\tau_{N} = N\tau_{0}$. The pulse was then extracted from the recurrent loop. The transmission intensity through the system was observed using the InGaAs avalanche photodiode D_1_ and recorded using a 600-MHz digital oscilloscope. The single round-trip intensity transmittance in the recurrent loop $T_{RL}$ was 0.07. These round-trip losses were compensated for when analyzing the experimental data. Figure 8b shows the configuration used for multiple quantum loophole interrogation. In this configuration, the pulse from the through port of the ring resonator was retuned in the recurrent loop. Switch S_2_ opened for $\Delta \tau$ = 1600 ns at $\tau_{m} = (m - 1)\tau_{0}$; otherwise, it was closed. The photon in the final port was observed as depicted in the trap interrogation experiment.

**Figure 9:**
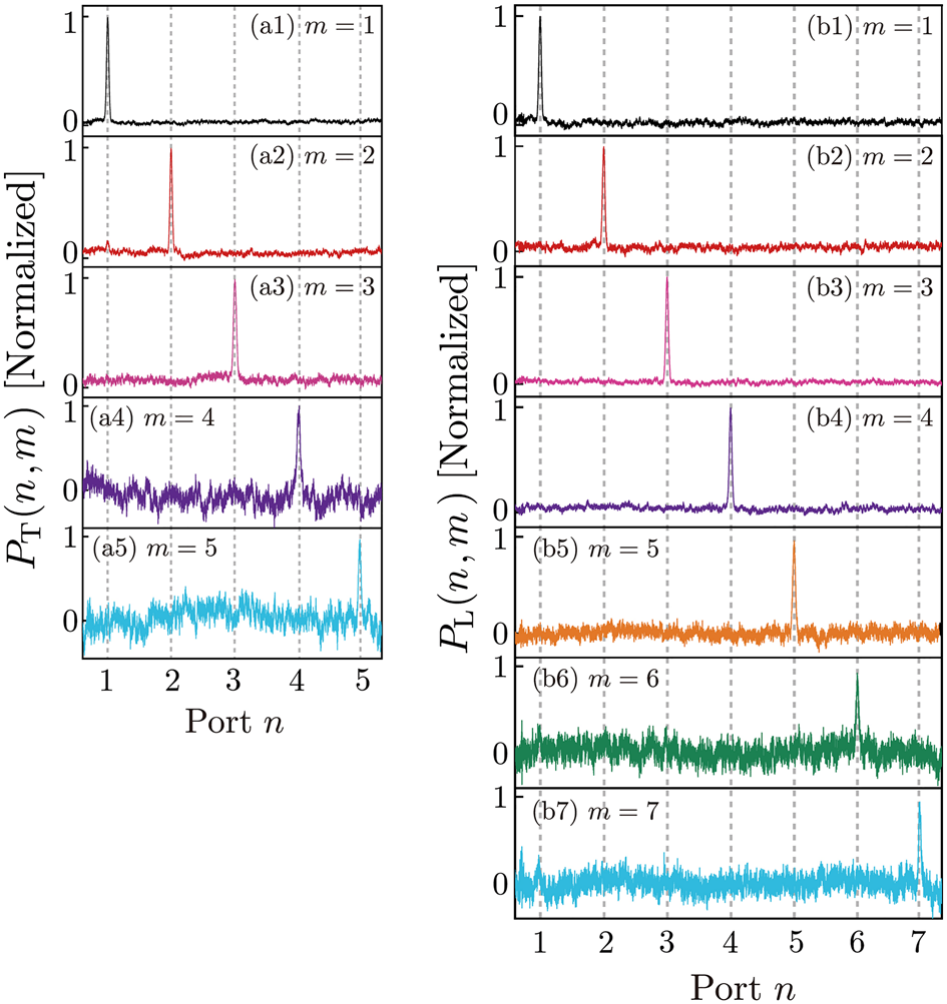
Transmission pulse intensities of the time-divided recurrent system. (**a**) Photon detection probability $P_{T}^{{}} (n,m)$ in the multiple quantum trap interrogation configuration with $N$ = 4 (four ring resonators). Horizontal axis shows the pulse passage time through a ring resonator, thus $n = 1 + t/\tau_{0}$, which corresponds to the number of exit ports $n$. Index $m$ represents the time at which S_2_ was closed, corresponding to the location of object $m$. (**a1**–**a4**) show the data for $m$ = 1–4, respectively. (**a5**) is the observation at the final port. (**b**) $P_{L}^{{}} (n,m)$ for the multiple quantum loophole interrogation configuration and $N$ = 6 (six ring resonators). Index $m$ represents the time at which S_2_ opened, corresponding to the location of loophole $m$. (**b1**–**b6**) Shows the data for $m$ = 1–6, respectively. (**b7**) is the observation at the final port. The parameters used in the experiments were as follows: $y_{2}$ = 0.95, $y_{1}$ = 0.78 ± 0.1 (finely adjusted to achieve the critical coupling condition) and $x_{1} x_{2}$ = 0.78 (− 1.1 dB).

Figure 9a shows the experimental results for $P_{T}^{{}} (n,m)$ based on the time-divided recurrent system of the multiple trap interrogation configuration. Measurements were carried out under the on-resonance frequency condition. The vertical axis is normalized. In the experiments, the pulse height was attenuated by a factor of $T_{RL} \times T_{Abs} (D)$ every time the interrogation stage increased. The horizontal axis is the real time $t$, but is labeled as the pulse passage time through the ring resonator $n = 1 + (t/\tau_{0} )$, which corresponds to the detection port number in Fig. 4a. Figure 9(a1) shows the results for $m$ = 1 (i.e., where S_2_ was closed for 1600 ns when the pulse initially arrived at the ring resonator). This corresponds to object existence at position $m$ = 1. In Fig. 4a, $P_{T}^{{}} (1,m = 1) = \sim 1$ and $P_{T}^{{}} (n \ne 1,m = 1) = \sim 0$; therefore, when the object was at position $m$ = 1, most photons were detected at port $n$ = 1. None were detected at port $n \ne$ 1. Figure 9(a2) shows the results for $m$ = 2; S_2_ was closed for 1600 ns when the pulse arrived in the ring resonator at $\tau_{2} = \tau_{0}$. In this case, most photons were detected at port $n$ = 2, thus $P_{T}^{{}} (2,m = 2) = \sim 1$. A small pulse was also detected at $\tau_{1} = 0$, thus $P_{T}^{{}} (1,m = 2) \ne 0$. These results indicate that the resonator was not in the perfect critical condition. Figure 9(a3) shows the results for $m$ = 3. Finally, Fig. 9(a5) shows the results when S_2_ was always open (i.e., when there was no object).

Figure 9b shows the $P_{L}^{{}} (n,m)$ results of the time-divided recurrent system in the multiple loophole interrogation configuration. In the loophole configuration, the output pulse from the through port was retuned in the recurrent loop. In our experiments, the absorption in the ring resonator was weak in the through port compared with the drop port ($T_{Pre} (C)$ > $T_{Abs} (D)$). Due to this larger transmission in the loophole configuration, we could explore the case of $N$ = 6. Figure 9(b1) shows the results for $m$ = 1; S_2_ was open for 1600 ns when the pulse initially arrived in the ring resonator. The pulse was detected by D_2_,$P_{L}^{{}} (1,m = 1) = \sim 1$, and $P_{L}^{{}} (n \ne 1,m = 1) = \sim 0$. Figure 9(b2) shows the results where S_2_ was open for 1600 ns when the pulse arrived in the ring resonator at $\tau_{2} = \tau_{0}$. In contrast to the trap configuration, nearly all photons were detected at $n$ = 2; $P_{L}^{{}} (2,m = 2) = \sim 1$ and $P_{L}^{{}} (n \ne 2,m = 2) = \sim 0$. Figure 9(b3)–(b6) show the results for $m$ = 3–6, respectively. $P_{L}^{{}} (n = m,m) = \sim 1$ and $P_{L}^{{}} (n \ne m,m) = \sim 0$ in all cases.

As the interrogation stage increased, the pulse exhibited severe attenuation in the recurrent system. To overcome this, our experiments were performed using pulses with the classical intensity level instead of the single photon level. However, destructive interference, which is the basis of the interaction-free measurement, occurs at the single photon level, as indicated by the single photon operator $\hat{a}^{ + } \hat{a}$ in Eqs. (5) and (7). Although the success rate was reduced considerably owing to the losses in the present system, the multiple interrogation worked for the critical coupling condition. The results obtained using strong classical light may thus be understood as the accumulation of results obtained using a single photon^17^. Therefore, the detection of photons at the presupposed ports supports the possibility of quantum multiple interrogation.

## Summary

We used quantum interaction-free measurements to determine where an object lies among multiple possible positions. Specifically, we explored multiple quantum trap and loophole interrogation using a serial array of add-drop ring resonators. We confirmed theoretically that it is possible to identify trap or loophole position with ~ 100% certainty. Using a dynamic recurrent loop system, we performed multiple trap and loophole interrogation experiments. Although our present experiments were performed using a strong pulse with a classical intensity level, our experiments adequately demonstrate the possibility of multiple quantum interrogations under the critical coupling condition.

It has been suggested that traditional quantum interrogation could have applications for the analysis of ultra-photosensitive molecules or detection of defects in ultra-photosensitive bio-systems^8^. This idea could be developed to achieve multiple quantum interrogation. Here, we used fiber ring resonators and the object was implemented within the fiber system. However, a similar system can be constructed using other constructs, such as an array of microspheres or micro-ring resonators coupled to bus waveguides, in which the adsorption of ultra-photosensitive materials on the microspheres or rings may be detected without real absorption of the probe photons.

## Data Availability

The datasets used and/or analyzed during the current study are available from the authors on reasonable request.
